# The Distribution of Complement Proteins in Soft and Hard Coronas Impacts Macrophage Uptake of Nanoparticles

**DOI:** 10.1002/adhm.202503534

**Published:** 2025-10-26

**Authors:** Ying Qiu, Tianchang He, Chunjie Miao, Xinyang Shi, Yuanyuan Niu, Volker Mailänder, Daniel Crespy, Katharina Landfester, Shuai Jiang

**Affiliations:** ^1^ Key Laboratory of Marine Drugs Chinese Ministry of Education School of Medicine and Pharmacy Ocean University of China Qingdao 266003 P. R. China; ^2^ Department of Dermatology University Medical Center of the Johannes Gutenberg‐University Langenbeckstr. 1 55131 Mainz Germany; ^3^ Department of Materials Science and Engineering School of Molecular Science and Engineering Vidyasirimedhi Institute of Science and Technology (VISTEC) Rayong 21210 Thailand; ^4^ Max Planck Institute for Polymer Research Ackermannweg 10 55128 Mainz Germany

**Keywords:** C3, macrophages, nano‐bio interactions, opsonization, soft corona

## Abstract

Complement proteins, as key constituents of the protein corona, facilitate the elimination of exogenous nanoparticles by immune cells, thereby limiting their tumor accumulation and therapeutic efficacy. Current research on complement proteins primarily focuses on the hard corona (HC), whereas their distribution and biological relevance in the soft corona (SC)—the dynamic layer directly interfacing with biological systems—remain underexplored. In this study, analyses in pooled and individual sera across different nanoparticle types, including carboxylated polystyrene nanoparticles (PS‐COOH), silica magnetic nanoparticles (SMNs), and liposomes (Lips), revealed that the third complement protein (C3) fragments associated with both HC and SC, with their distribution dictated by nanoparticle surface chemistry. For PS‐COOH and SMNs, iC3b is preferentially enriched in the SC, whereas Lips surface favored iC3b deposition within the HC, likely due to exposed amino groups that facilitate C3 attachment via thioester‐amine reactions. Functional assays demonstrate that macrophage uptake is primarily driven by HC‐bound iC3b, while SC proteins attenuate this process by shielding HC‐bound C3 fragments and limiting their accessibility to complement receptors. Moreover, SC does not effectively mediate nanoparticle‐phagocyte interactions, as its dynamic nature prevents stable engagement with cellular receptors. Collectively, these findings provide new mechanistic insights into SC‐mediated clearance of nanomedicines.

## Graphical abstract

Protein corona plays a critical role in the biological behavior of nanoparticles. This study investigates the distribution of complement proteins in soft corona and their impact on the macrophage uptake of polymer nanoparticles. It underlines the significance of soft corona investigation for understanding in vivo performance of nanomedicines.

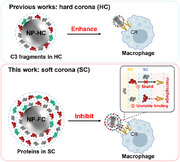

## 1 Introduction

Nanoparticles (NPs) for drug delivery are designed to selectively enhance drug accumulation in tumor sites. However, a mere 0.7% (median) of NPs administered intravenously were found to effectively reach solid tumors.^[^ ^1^ ^]^ The majority of NPs are sequestered by mononuclear macrophage system in the liver and spleen. Indeed, resident macrophages recognize NPs via opsonins present on their surface upon contact with blood.^[^ ^2^, ^3^ ^]^ Once entering the bloodstream, NPs trigger the complement cascade through 3 pathways: classic pathway, lectin pathway, and alternative pathway.^[^ ^4^, ^5^, ^6^ ^]^ Upon activation, all complement pathways form C3 convertase, which rapidly cleaves the third complement protein (C3) into functional effector molecules, including C3a and C3b. The latter is further deposited on NPs and assembles to form C5 convertase, which in turn cleaves the fifth complement protein (C5) into C5a and C5b. Finally, C5b combines with complement proteins C6, C7, C8, and C9, forming terminal membrane attack complex (MAC or C5b‐9). The complement activation products hinder the clinical translation of NPs. C3a and C5a are closely associated with hypersensitivity reactions during NP administration, while the MAC can lead to premature release of the encapsulated drug.^[^ ^7^, ^8^, ^9^, ^10^, ^11^, ^12^, ^13^, ^14^, ^15^ ^]^ More importantly, the C3b covalently binds to NPs, facilitating their clearance by phagocytes, a process called opsonization.^[^ ^16^, ^17^ ^]^ Studies have shown a positive correlation between the abundance of C3 adsorbed on NPs and their uptake by macrophages.^[^ ^18^, ^19^ ^]^ This is also exemplified in the accelerated blood clearance phenomenon, where complement activation and C3 opsonization promote the removal of PEGylated NPs by Kupffer cells.^[^ ^9^, ^20^ ^]^

Plasma proteins adsorb onto NPs, giving rise to a protein corona (PC) that comprises both hard corona (HC) and soft corona (SC) proteins. The HC and SC represent two distinct layers of the PC with fundamentally different binding behaviors. HC proteins bind to NPs with high affinity and remain stably attached for longer durations, whereas SC proteins associate via weak, reversible interactions and undergo rapid exchange with proteins in the surrounding milieu.^[^ ^21^, ^22^, ^23^ ^]^ These binding characteristics mean that the composition of PC is strongly influenced by NP physicochemical properties and environmental conditions, determining the biological relevance of SC and HC in regulating NP fate.^[^ ^23^, ^24^, ^25^ ^]^ Importantly, while HC has traditionally been emphasized because of its stability and persistence, growing evidence indicates that the SC, as the outermost and most dynamic layer directly interfacing with the biological environment, plays an indispensable role in NP recognition, opsonization, and cellular uptake.^[^ ^26^, ^27^, ^28^, ^29^ ^]^ Current research methodologies often employ a separation procedure, such as repetitive centrifugation, to isolate NP‐PC complexes. However, these approaches may lead to the loss of loosely bound SC proteins, resulting in a predominant focus on elucidating the composition and biological impacts of HC proteins.^[^ ^18^, ^19^, ^30^ ^]^ Therefore, the crucial role of outer‐layer SC proteins has been overlooked. However, proteins within the SC, including deposited C3, are believed to exert a predominant influence on the recognition and clearance of NPs by macrophages.

To amplify the complement cascade and opsonize exogenous particles, the produced C3b typically binds covalently to amino or hydroxyl groups on particle surfaces.^[^ ^31^ ^]^ Thus, the PC can participate in complement activation by providing recognition sites for C3b, owing to the abundant amino groups in the corona proteins. For instance, adsorbed immunoglobulins on NPs have been reported to promote complement activation and C3 opsonization.^[^ ^5^, ^32^, ^33^ ^]^ Chen et al.^[^ ^34^ ^]^ demonstrated that nearly all C3 molecules were located around the NP surface through binding with the PC. Furthermore, their study unveiled a dynamic and reversible binding of C3 to the PC of NPs, implying the potential presence of C3 in the SC. Therefore, achieving a comprehensive determination of complement protein distribution in the PC and studying its impact on NP uptake by macrophages is essential for understanding the in vivo clearance mechanisms of NPs.

**Scheme 1:**
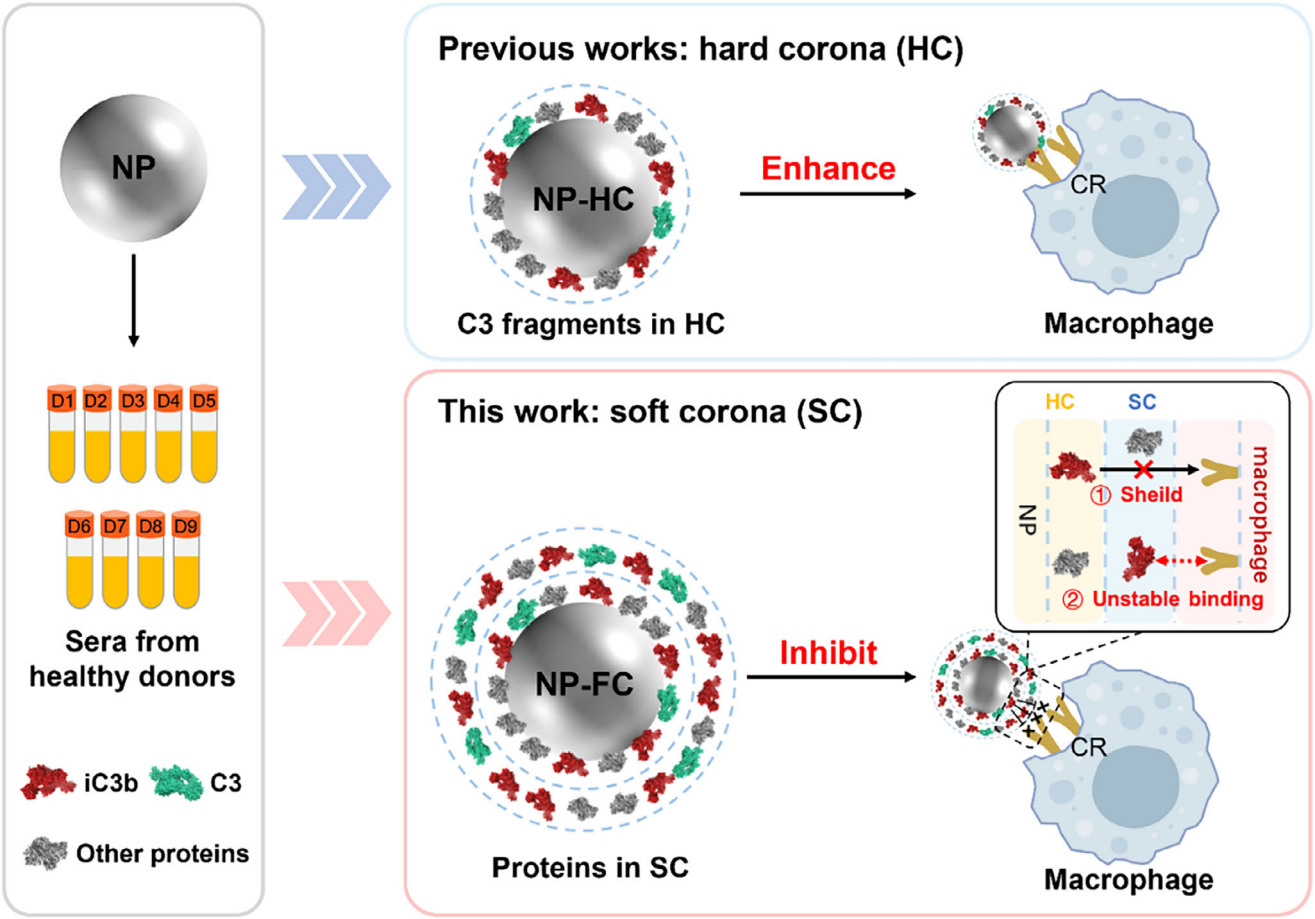
Schematic illustration of SC and HC in regulating complement‐dependent NP‐macrophage interactions. Macrophage uptake is primarily driven by HC‐bound iC3b, whereas SC proteins attenuate this process by shielding HC‐bound fragments and, due to their dynamic nature, fail to stably engage complement receptors (CRs).

In this study, we elucidated the distribution of C3 fragments in SC and HC and its impact on the macrophage uptake of polymer NPs (**Scheme** **1**). Quantification of C3 fragments in the PC was performed through dot blot and western blot experiments. EDTA and zymosan (Zymo) were used to further study the adsorption of C3 fragments in SC and HC. Biological effects of C3 fragments were explored through cellular uptake experiments with macrophages from human sources and murine (differentiated THP‐1 (dTHP‐1) and RAW264.7). To account for inter‐individual variability and NP specificity, experiments were extended to sera from nine individual donors as well as two clinically relevant nanomaterials: silica magnetic nanoparticles (SMNs) and liposomes (Lips). Our work unveils the distribution of C3 proteins within the PC and their consequential impact on macrophage clearance, underlining the significance of SC investigation for understanding and predicting in vivo performance of nanomedicines.

## 2 Results and Discussion

### 2.1 Distribution of C3 Fragments in SC and HC

**Figure 1:**
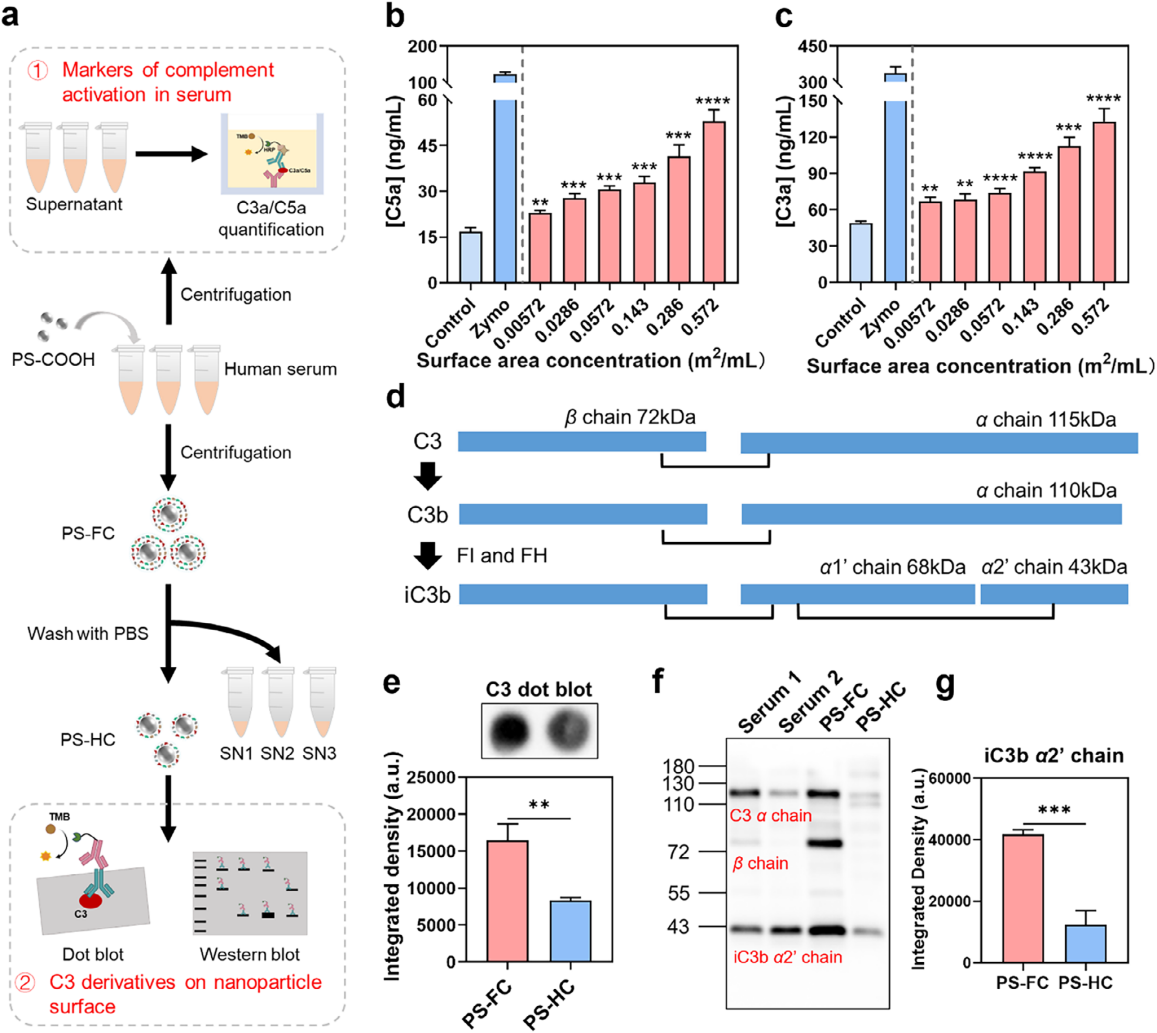
Analysis of C3 fragments in FC and HC of PS‐COOH. a) Schematic workflow for analyzing complement activation and C3 deposition of PS‐COOH. Concentration of C5a (b) and C3a (c) in pooled human serum after incubation of PS‐COOH at various surface area concentrations (0.00572–0.572 m^2^ mL^−1^). Water served as the negative control, while Zymo (1 mg mL^−1^) was the positive control. d) Schematic representation of C3 activation and processing. Native C3 consists of a *β* chain (≈72 kDa) and an *α* chain (≈115 kDa). Upon activation, C3 is cleaved into C3b, in which the *α* chain appears at ≈110 kDa. C3b is further processed by factor I and factor H, into iC3b. In iC3b, the *α* chain is composed of two characteristic fragments: *α*1’ (≈68 kDa) and *α*2’ (≈43 kDa). e) Detection of C3 fragments deposition on PS‐COOH surface using dot blot. f) Western blot analysis of C3 fragments in FC and HC. Bands corresponding to C3 *α* chain (≈115 kDa), iC3b *α*2’ chain (≈43 kDa), and *β* chain (≈72 kDa). Serum 1 represented serum after PBS treatment. Serum 2 represented serum after Zymo treatment, which induces strong complement activation. g) Quantification of band of iC3b *α*2’ chain (43 kDa) using image J. Data are presented as the mean ± standard deviation (n = 3). **p < 0.01, ***p < 0.001, ****p < 0.0001.

Polystyrene nanoparticles are widely used as a classical model for studying PC formation owing to their physicochemical stability and facile separation. Here, we employed carboxylated polystyrene nanoparticles (PS‐COOH) to investigate the adsorption of C3 fragments in the SC and HC. Dynamic light scattering (DLS) analysis revealed a hydrodynamic size was ≈155 nm with a PDI of 0.168 (Table S1, Supporting Information), and transmission electron microscopy (TEM) confirmed their uniform spherical morphology (Figure S1, Supporting Information). First, we assessed their complement activation in pooled human serum. PS‐COOH at increasing surface area concentrations was incubated with serum, and the levels of complement activation markers C3a and C5a were quantified by enzyme‐linked immunosorbent assay (ELISA) (**Figure** **1**a). The results showed a dose‐dependent elevation of C3a and C5a, inducing ≈2.7‐ and ≈3.1‐fold increases at 0.572 m^2^ mL^−1^ compared with the negative control (Figure 1b,c). Complement activation was further verified by a hemolytic CH50 assay. The results showed that 0.572 m^2^ mL^−1^ PS‐COOH reduced CH50 from 83.4 to 26.5 U mL^−1^ (Figure S2b,c, Supporting Information). In comparison, ELISA results showed that PS‐COOH was activating the complement system, even at a concentration of 0.00572 m^2^ mL^−1^, underscoring that ELISA assay is more sensitive compared with the hemolytic assay. Collectively, these results established PS‐COOH as a robust complement activator and provided the foundation for subsequent analysis of C3 fragment distribution between the SC and HC.

To characterize C3 adsorption on PS‐COOH, NP‐serum incubation mixtures were centrifuged once to prepare the full corona for PS‐COOH (PS‐FC), which was composed of SC and HC. Then the PS‐FC was washed three times with PBS to obtain PS‐COOH with HC (PS‐HC). Adsorbed proteins were then desorbed using 2 wt.% SDS, yielding FC and HC solutions. Bicinchoninic acid (BCA) and western blot analyses confirmed that negligible proteins remained on the desorbed PS‐COOH surface, confirming effective recovery of corona proteins (Figure S3a,b, Supporting Information).

Protein quantification showed higher protein concentrations in the FC compared to the HC, with abundant proteins also detected in the washing supernatants, especially the first washing supernatant (SN1), indicating the predominant presence of SC proteins within SN1 (Figure S3e, Supporting Information). The sodium dodecyl sulfate‐polyacrylamide gel electrophoresis (SDS‐PAGE) revealed several enriched bands in the SC (≈45, 100, 180, and 245 kDa) relative to the HC (Figure S3f, Supporting Information). Among these, the difference at 45 kDa was the most pronounced. Given that C3 activation generates fragments such as C3b (*α* chain, ≈105 kDa) and iC3b (*α*2’ chain, ≈43 kDa), these enriched bands likely correspond to complement activation products.

We next investigated whether these SC‐associated proteins included C3 fragments. Dot blot results revealed the presence of abundant C3 fragments in both FC and HC, with higher levels in FC, suggesting the presence of C3 fragments in the SC (Figure 3d). Importantly, dot blot cannot distinguish between intact C3 and its activation products, yet only specific forms of C3 (such as C3b, iC3b, and C3dg) are biologically active in opsonization and recognized by complement receptors (CRs) on macrophages. Native C3 is composed of a *β* chain (≈72 kDa) and an *α* chain (≈115 kDa) (Figure 1d). Upon activation, C3 is cleaved into C3b, in which the *α* chain appears at ≈110 kDa. Further proteolysis of C3b by factor I and factor H generates iC3b, where the *α* chain is split into two fragments: *α*1’ (≈68 kDa) and *α*2’ (≈43 kDa). Therefore, additional western blot analysis was conducted to identify specific C3 fragments. Distinct bands corresponding to the C3 *α* chain (≈115 kDa), *β* chain (≈72 kDa), and iC3b *α*2’ chain (≈43 kDa) were detected (Figure 1f), confirming adsorption of both native C3 and activated iC3b. Water‐treated serum was used as negative control, while Zymo‐treated serum served as positive control. Following Zymo treatment, a decrease in C3 *α* chain band intensity and an increase in iC3b *α*2’ chain band intensity were observed, indicating activation of the complement system by Zymo and high complement activity of the serum (Figure 1e). Importantly, quantitative analysis revealed that FC contained ≈3.4‐fold more iC3b than HC, highlighting preferential enrichment of opsonin iC3b within the SC (Figure 1f,g).

We further analyzed C3 fragments in the washing supernatants by western blot, as most SC proteins were released during washing. Consistent with the SDS‐PAGE, the majority of iC3b was detected in SN1, with levels slightly higher than those in the HC, confirming that substantial iC3b were associated with the SC of PS‐COOH.

**Figure 2:**
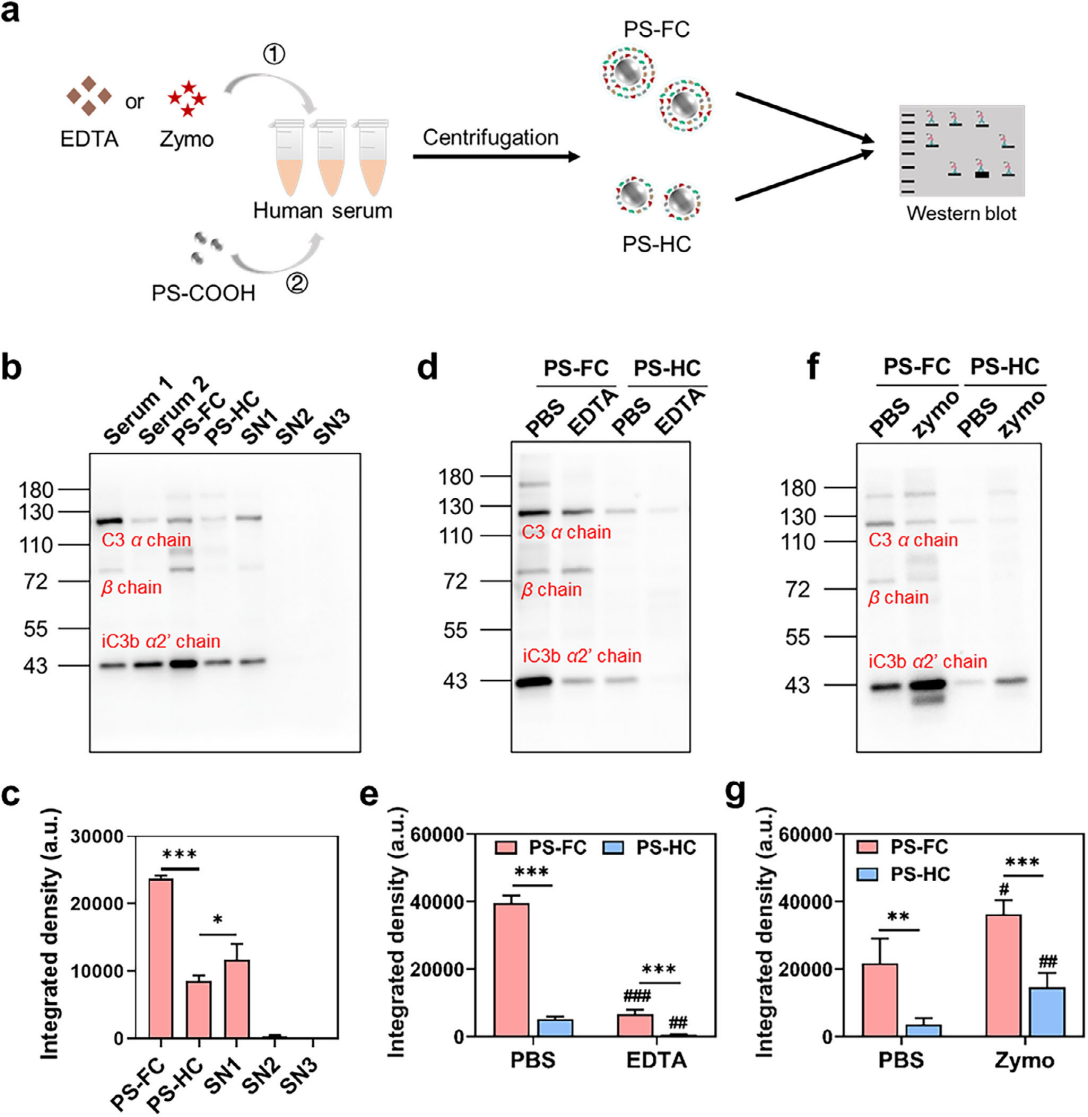
Complement activation‐dependent deposition of iC3b in FC and HC. a) Scheme illustrating the preparation of PS‐FC and PS‐HC from the serum pre‐treated with EDTA or Zymo. b) Western blot analysis of C3 fragments in FC, HC, and washing supernatants. c) Quantitative gray value of band of iC3b *α*2’ chain analyzed using image J. Western blot analysis and quantitative gray value of band of iC3b *α*2’ chain adsorbed on PS‐COOH after incubation with EDTA‐treated serum (d,e) or Zymo‐treated serum (f,g). FC and HC prepared from PBS‐treated serum served as controls. Equivalent amounts of PS‐COOH were used to compare the adsorbed C3 fragments in FC and HC. Data are presented as mean ± standard deviation (n = 3). *p < 0.05, **p < 0.01, ***p < 0.001 (comparison between PS‐FC and PS‐HC within the same treatment); **^##^** *p* < 0.01, **^###^** *p* < 0.001 (comparison among PBS‐, EDTA‐, and Zymo‐treated groups within PS‐FC or PS‐HC).

Given that PS‐COOH strongly activated complement, we next assessed how complement activation influenced C3 distribution in SC and HC (**Figure** **2**a). When PS‐COOH was incubated with EDTA‐treated serum, the western blot revealed markedly reduced iC3b in both FC and HC (Figure 2d,e). Grayscale quantification indicated ≈85% decrease in iC3b concentrations in SC and ≈85% decrease in HC, indicating that complement activation generating iC3b is present in both SC and HC.

Zymo can activate the alternative pathway and is usually used for positive control of study in complement activation.^[^ ^35^ ^]^ We further increased the amounts of C3 fragments in serum by Zymo to study their adsorption in SC and HC. Zymo was incubated with serum to generate sufficient amounts of iC3b, and PS‐COOH was then exposed to this serum to prepare FC and HC. Western blot analysis showed a significant increase in the amounts of adsorbed iC3b in both FC and HC (Figure 2f,g). The iC3b concentrations increased by ≈20% in SC and ≈308% in HC. However, its level in the FC was ≈2.5‐fold higher than in the HC, confirming that the SC still contained more iC3b than the HC. These results confirmed that iC3b in serum were bound to both SC and HC, though primarily to HC.

### 2.2 Increased Cellular Uptake of PS‐COOH upon Adsorption of C3 Fragments

**Figure 3:**
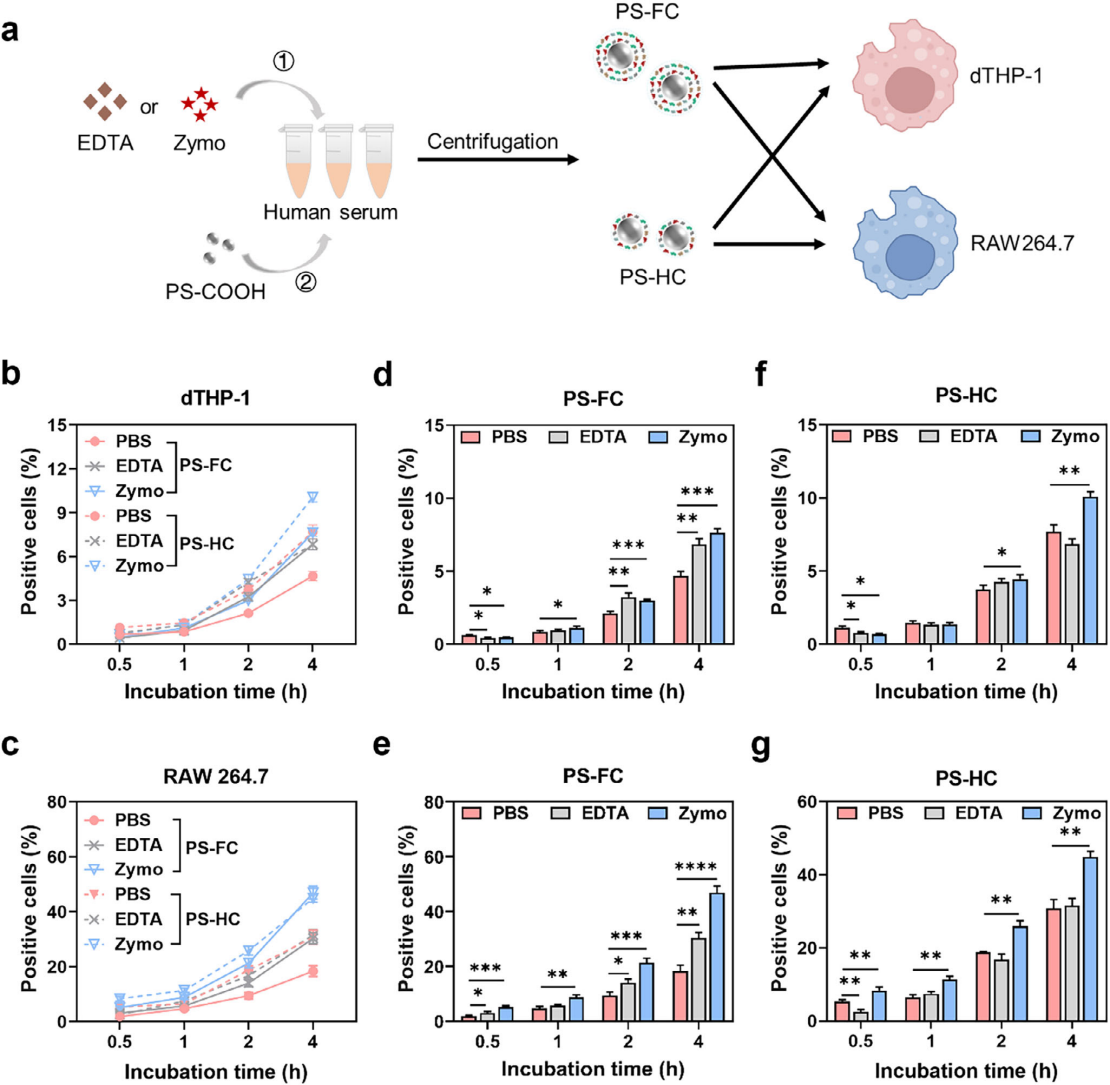
Impact of C3 fragments in FC and HC on cellular uptake of NPs. a) Scheme illustrating PS‐FC and PS‐HC exposed to dTHP‐1 and RAW264.7. PS‐FC and PS‐HC were prepared by incubating PS‐COOH with serum pre‐treated with PBS, EDTA, or Zymo. The dTHP‐1 and RAW264.7 were then exposed to PS‐FC and PS‐HC (50 µg mL^−1^) for 0.5, 1, 2, and 4 h. Comparison of cellular uptake of PS‐PC complexes prepared from sera after different treatments by dTHP‐1 (b) and RAW264.7 (c). Cellular uptake of PS‐FC and PS‐HC by dTHP‐1 (d,f) and RAW264.7 (e,g). Data are presented as mean ± standard deviation (n = 3). *p < 0.05, **p < 0.01, ***p < 0.001, ****p < 0.0001.

Complement proteins, especially opsonin C3, enhance macrophage uptake of NPs via binding with CRs on macrophage surfaces.^[^ ^36^ ^]^ Increasing evidence indicates that SC proteins play a crucial role in regulating the biological behaviors of NPs.^[^ ^26^, ^27^ ^]^ To validate the influence of C3 fragments in the SC on NP‐macrophage interactions, we conducted cellular uptake experiments using differentiated human monocytic THP‐1 and mouse‐derived macrophages RAW264.7 (**Figure** **3**a).

First, PS‐COOH was incubated with serum to prepare PS‐FC and PS‐HC, which were then incubated with dTHP‐1 and RAW264.7 cells for 0.5, 1, 2, or 4 h. Results showed that the uptake of both PS‐FC and PS‐HC by dTHP‐1 cells increased with prolonged incubation time (Figure 3b). Notably, PS‐HC exhibited a higher uptake than PS‐FC. Similar results were obtained with RAW264.7 (Figure 3c). These findings suggested that C3 fragments within the SC may not directly mediate NP‐phagocyte interactions. Instead, it may interfere with the accessibility of HC‐associated C3 fragments to cellular CRs. Moreover, the SC proteins did not effectively mediate NP‐phagocyte interactions, as their dynamic and exchangeable nature prevents stable engagement with cellular receptors.

To further investigate the impact of SC‐associated C3 fragments on macrophage uptake, serum was pre‐treated with EDTA before preparing PS‐FC and PS‐HC. Compared to the PBS‐treated group, the uptake of PS‐HC by dTHP‐1 and RAW264.7 remained unchanged, whereas PS‐FC uptake increased (Figure 3d–g). Notably, the reduction of C3 fragments in PS‐FC and PS‐HC did not lead to decreased macrophage uptake. For PS‐HC, the lack of reduced macrophage uptake may be attributed to the relatively low adsorption of iC3b, which had a minimal influence on cellular uptake. Previous studies have demonstrated that the amount of C3 on NPs was a key determinant of hepatic clearance. Nagayama et al.^[^ ^37^ ^]^ reported that prolonged serum incubation (360 min) led to enhanced C3 adsorption and increased hepatic uptake. In contrast, NPs incubated in heat‐treated serum, where complement proteins were deactivated, exhibited reduced liver clearance only at 360 min, underscoring the critical role of C3 levels in regulating NP‐macrophage interaction. C3 fragments adsorb to NP surface by binding to immunoglobulin G and other serum proteins on the NPs.^[^ ^34^ ^]^ EDTA treatment reduced C3 fragment adsorption and likely altered the composition of the outermost protein layer. This modification could lead to enhanced interaction between opsonins, such as immunoglobulin G, and macrophages, potentially explaining the increased uptake of PS‐FC following EDTA pre‐treatment.

Moreover, dTHP‐1 and RAW264.7 were incubated with PS‐FC and PS‐HC prepared from Zymo‐treated serum for varying durations. Results showed that uptake of PS‐FC and PS‐HC by dTHP‐1 increased compared to the PBS‐treated group, with higher uptake by PS‐FC (Figure 3d,f). For RAW264.7, similar results were observed, but the uptake of PS‐HC was comparable to PS‐FC (Figure 3e,g).

To complement flow cytometry, we performed confocal laser scanning microscopy (CLSM), which confirmed higher uptake of PS‐HC than PS‐FC by dTHP‐1 cells at 4 h (Figure S5, Supporting Information). Zymo pretreatment of serum slightly increased uptake of both, but PS‐HC uptake remained higher. Co‐staining with Lysotracker showed that most internalized particles localized to endo/lysosomes, indicating true internalization rather than surface binding. Collectively, these findings further confirmed that C3 fragments within the SC may not promote NP‐phagocyte interactions.

### 2.3 Time‐Dependent Adsorption of C3 Fragments in the FC and HC of PS‐COOH

**Figure 4:**
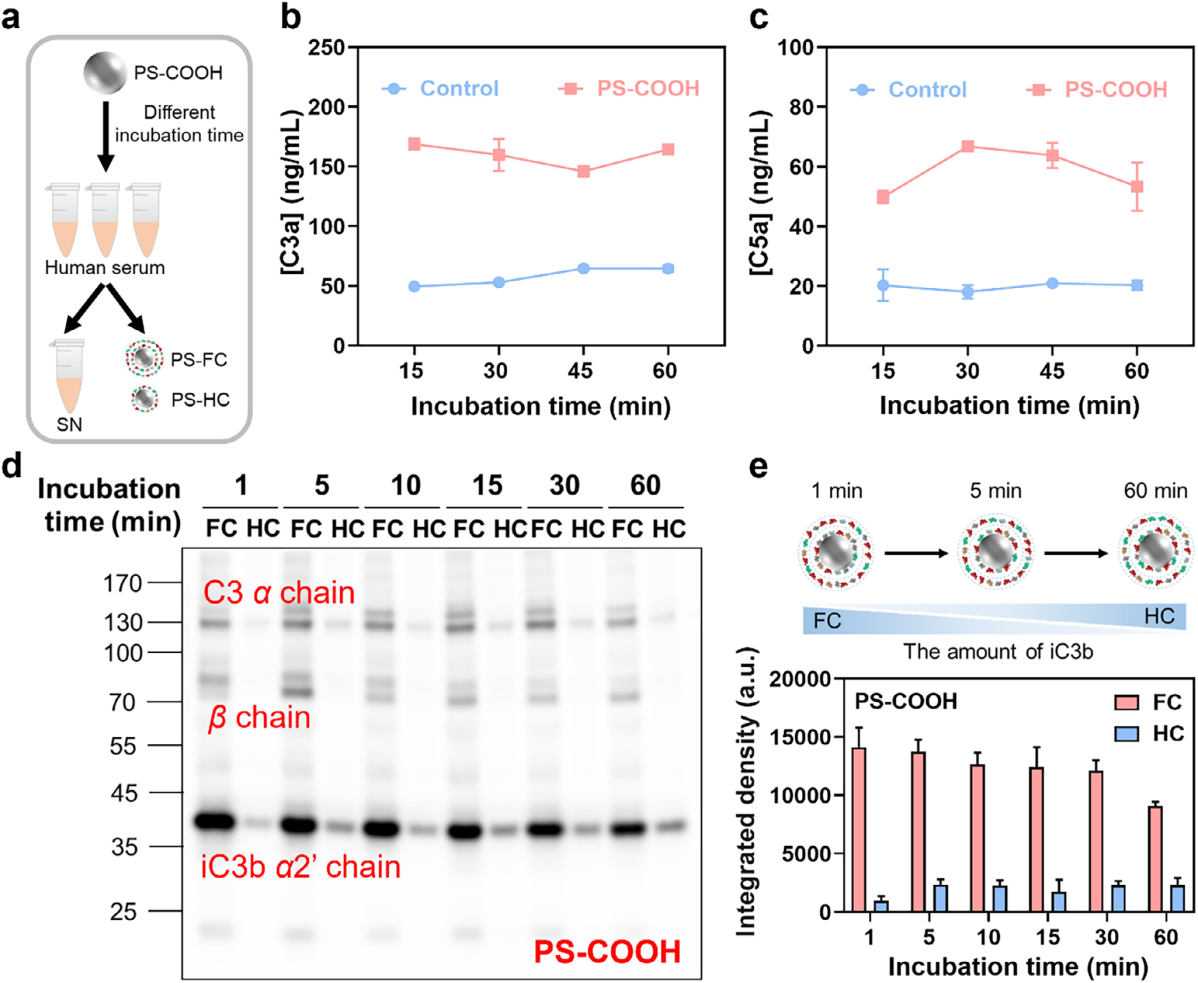
Time‐dependent adsorption of C3 fragments in the FC and HC of PS‐COOH. a) Scheme illustrating the preparation of FC and HC. Concentrations of C3a (b) and C5a (c) in human serum after incubation of PS‐COOH for 15, 30, 45, and 60 min. The surface area concentration of NPs in the incubation mixtures was 0.143 m^2^ mL^−1^. d) Western blot analysis of C3 fragments in FC and HC at different incubation times (1, 5, 10, 15, 30, and 60 min). e) Grayscale quantification of iC3b *α*2’ chain (≈43 kDa). Data are presented as mean ± standard deviation (n = 3).

We further investigated the influence of incubation time on complement activation (**Figure** **4**a). Incubating PS‐COOH in pooled human serum for 15–60 min showed a stable C3a concentration (Figure 4b), signifying its rapid generation, while C5a concentration notably increased with extended incubation durations (Figure 4c). The alternative pathway, constituting approximately 80–90% of total complement activation, amplifies complement response on exogenous particles using C3b produced with the 2 other pathways to assemble C3 convertase. This amplification involves initially increasing the local concentration of C3b on NPs, followed by the gradual association of accumulated C3b with C3 convertases to form C5 convertase, which cleaves C5 into C5a and C5b.^[^ ^31^ ^]^ Consequently, this mechanism may account for the rapid generation of C3a within 15 min, while C5a concentration continued to rise until 45 min.

Next, the time‐dependent adsorption of C3 fragments on PS‐COOH was investigated. PS‐COOH was incubated with serum for 1, 5, 10, 15, 30, and 60 min, and C3 fragments in FC and HC were analyzed by western blot. As early as 1 min of incubation, a substantial amount of iC3b was already detected on the PS‐COOH surface, with its level in the FC being ≈15.0‐fold higher than that in the HC, indicating pronounced enrichment of iC3b within the SC at early time point (Figure 4d,e). With prolonged incubation to 5 min, iC3b in the HC increased to ≈2.5‐fold of that at 1 min and remained stable thereafter, while the iC3b level in the FC gradually declined. After 60 min, the overall iC3b abundance in the FC decreased by 37.8% relative to 1 min, yet still remained ≈3.9‐fold higher than that in the HC. These findings suggested that complement activation occurred rapidly (<1 min), with nascent C3b rapidly inactivated to iC3b under the regulation of factor H and factor I. A fraction of iC3b subsequently stabilized in the HC, while another fraction underwent further proteolytic processing into C3dg and C3d. The gradual loss of the iC3b in the FC was consistent with this turnover. Notably, although C3d/C3dg are considered the predominant C3 derivatives deposited on NPs in vivo, no clear bands were detected here, likely due to incorporation into high‐molecular‐weight complexes that masked the expected ≈37 kDa position.^[^ ^38^ ^]^ Collectively, our results demonstrated that PS‐COOH can rapidly trigger complement activation in vitro and acquire dynamically evolving patterns of C3 fragments on their surface.

### 2.4 Inter‐Individual Variability and NP‐Dependent Complement Activation, C3 Adsorption, and Macrophage Uptake

**Figure 5:**
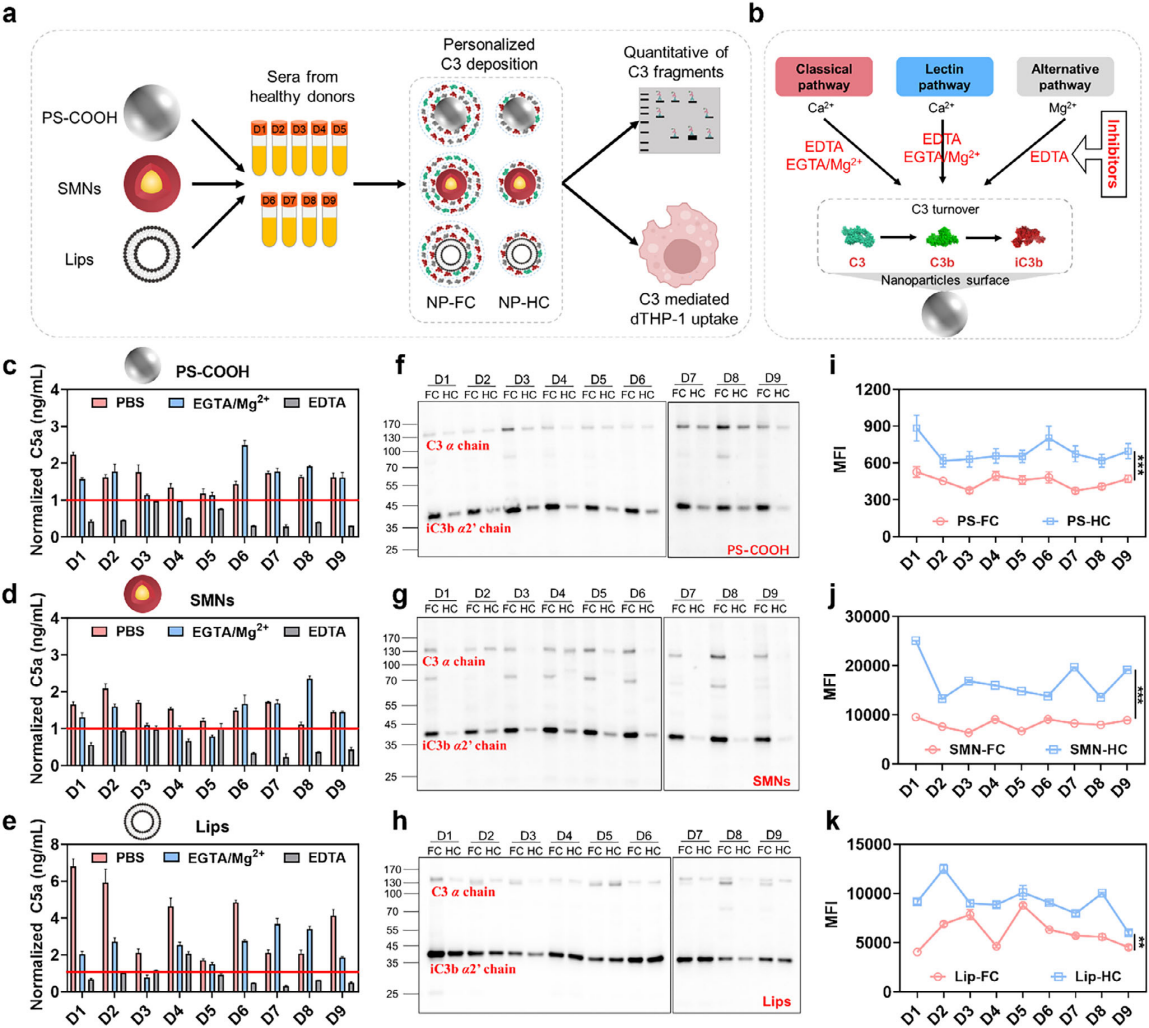
Complement activation, C3 adsorption, and macrophage uptake of three NP types across individual human sera. a) Schematic workflow for assessing NP‐induced complement activation, C3 adsorption, and macrophage uptake. b) Diagram of complement activation pathways on NP surfaces and their selective inhibition. Alternative pathway remains functional in the presence of 10 mm EGTA/Mg^2+^, whereas Ca^2+^‐sensitive pathways (classic and lectin) are inhibited. All pathways are fully inhibited by 10 mm EDTA. Complement activation and pathway analysis in sera from nine donors for PS‐COOH (c), SMNs (d), and Lips (e). The reported concentrations are normalized to baseline levels in serum without NPs, as shown by the solid line in each figure. Western blot analysis of C3 fragment adsorption in the FC and HC of PS‐COOH (f), SMNs (g), and Lips (h). Uptake of FC‐ and HC‐coated PS‐COOH (i), SMNs (j), and Lips (k) by dTHP‐1 cells. Data are presented as mean ± standard deviation (n = 3). **p < 0.01, ***p < 0.001.

To better approximate clinical scenarios, we further investigated complement activation, C3 fragment adsorption, and macrophage uptake of PS‐COOH in nine individual human sera (**Figure** **5**a). The use of sera from multiple donors ensured that the observed effects were not serum‐specific but generalizable across individual variations. Two clinically relevant NP formulations were also included: SMNs (applicable for magnetic resonance imaging) and Lips (the most widely used nanocarriers with multiple approved formulations). DLS showed hydrodynamic sizes of ≈125 nm for SMNs and ≈127 nm for Lips, with PDIs of 0.130 and 0.219, respectively (Table S1, Supporting Information). TEM confirmed their uniform spherical morphology (Figure S1, Supporting Information).

Given the strong impact of NP size on complement activation, we normalized concentrations by surface area and incubated equal surface areas of the three NPs with nine sera, quantifying C5a in supernatants. Baseline differences of C5a levels across individuals were corrected by normalizing to PBS‐treated serum. PS‐COOH and SMNs significantly elevated C5a levels in eight donors, with mean increases of ≈1.62‐ and ≈1.52‐fold, respectively (Figure 5c,d). Remarkably, Lips induced the strongest complement activation, increasing C5a levels in all donors by an average of ≈3.81‐fold (Figure 5e). Given that Lips contained 1,2‐distearoyl‐*sn*‐glycero‐3‐phosphocholine (DSPC), 1,2‐distearoyl‐*sn*‐glycero‐3‐phosphoethanolamine (DSPE), and cholesterol, the partially exposed primary amine groups of DSPE may account for this enhanced activation, consistent with prior studies.^[^ ^5^, ^31^, ^32^ ^]^

To dissect the pathways involved, we employed complement inhibitors. EDTA, which blocks all pathways by chelating Ca^2^⁺ and Mg^2^⁺, completely suppressed activation for all NP types (Figure 5c–e). In contrast, EGTA/Mg^2^⁺, which blocks Ca^2^⁺‐dependent classical and lectin pathways but allows the alternative pathway to proceed, had little effect on PS‐COOH and SMNs, and even occasionally enhanced C5a levels, indicating predominant activation via the alternative pathway (Figure 5c,d). For Lips, EGTA/Mg^2^⁺ treatment led to an average ≈49% reduction in C5a levels across most donor sera, but in donor 7 (D7) and D8 still increased (Figure 5e). It suggested that Lips‐induced complement activation involved both Ca^2^⁺‐dependent (classical or/and lectin) and alternative pathways.

Complement activation in pooled serum mirrored individual results, with C5a levels rising ≈1.5‐, 1.7‐, and 4.3‐fold for PS‐COOH, SMNs, and Lips, respectively, compared to PBS‐treated serum (Figure S5, Supporting Information). Moreover, EGTA/Mg^2^⁺ had little effect on PS‐COOH and SMNs but significantly inhibited Lips, indicating predominant activation via the alternative pathway for PS‐COOH and SMNs in pooled serum, whereas Lips engaged both Ca^2^⁺‐dependent and alternative pathways. Although there was inter‐individual variation in complement proteins and regulators, assays using pooled serum still captured representative complement activation patterns of NPs.

Subsequently, the adsorption of C3 fragments in the SC and HC was also studied. The results showed that the complement activation fragment iC3b (*α*2’ chain, ≈43 kDa) was detected in both the FC and HC for three types of NPs. For PS‐COOH and SMNs, iC3b levels in the FC were ≈2.7‐ and ≈3.9‐fold higher than in the HC, respectively, thereby indicating a dominant contribution of SC‐associated iC3b (Figure 5f,g). In contrast, for Lips, FC contained only ≈1–1.5‐fold more iC3b than HC, suggesting that iC3b was preferentially deposited in the HC (Figure 5h). C3 fragments predominantly attach to NP surfaces through thioester‐mediated covalent interactions with nucleophilic groups such as hydroxyls and amines.^[^ ^31^, ^34^ ^]^ Compared with PS‐COOH and SMNs, the presence of exposed amino groups on the surface of Lips (derived from DSPE) may favor iC3b deposition within the HC. This mechanistic difference likely explained the distinct distribution patterns of iC3b among the three NP types. Collectively, these results indicated that, consistent with PS‐COOH, both SMNs and Lips recruited substantial amounts of C3 activation fragments into their SC.

Finally, we investigated the role of C3 activation fragments in mediating uptake of NPs by dTHP‐1 macrophages. Importantly, dTHP‐1 uptake experiments revealed a consistent trend across all NP types: uptake of FC‐coated NPs was significantly lower than that of HC‐coated NPs (Figure 5i–k). These findings suggested that SC proteins may attenuate uptake by masking C3 fragments associated with the HC, thereby reducing their accessibility to CRs. Moreover, the SC did not effectively mediate NP‐phagocyte interactions, as its dynamic and exchangeable nature prevented stable engagement with cellular receptors.

## 3 Conclusion

In this study, we investigated the distribution of C3 fragments in the SC and HC of NPs and their impact on NP‐immune cell interactions. Our analyses revealed that the distribution of C3 fragments was dependent on the surface chemistry of NPs. For PS‐COOH and SMNs, iC3b was preferentially enriched in the SC, whereas Lips surface favored iC3b deposition within the HC, likely due to exposed amino groups that facilitated C3 attachment via thioester‐amine reactions. Importantly, the macrophage uptake of NPs was primarily governed by HC‐bound C3 fragments, whereas SC proteins may attenuate uptake by masking C3 fragments associated with the HC, thereby reducing their accessibility to CRs. Moreover, the SC did not effectively mediate NP‐phagocyte interactions, as its dynamic and exchangeable nature prevented stable engagement with cellular receptors. These results highlight the central role of the SC in PC biology and provide new guidance for designing nanomedicines with improved immune evasion.

Notably, while in vitro incubation provides well‐controlled settings, the in vivo environment is far more complex and dynamic, where blood flow, fluid composition, and immune responses, et al. continuously remodel the corona.^[^ ^24^, ^39^ ^]^ Corona composition has been shown to vary markedly across tissues: albumin and immunoglobulin G are enriched in the corona formed in liver, where extracellular matrix proteins dominate in the corona in tumor tissue.^[^ ^40^ ^]^ Complement activation and C3 opsonization are likewise affected by these dynamic changes. For example, Chen et al.^[^ ^34^ ^]^ reported that C3 pre‐adsorbed on superparamagnetic iron oxide nanoworms rapidly decreased after injection into mice, suggesting active C3 exchange in vivo. In a follow‐up study, PEG‐antibody functionalized nanoworms showed significant C3 deposition and high complement‐dependent uptake by leukocytes in vivo (mice), but far weaker C3 deposition and macrophage uptake in vitro.^[^ ^41^ ^]^ Therefore, the dynamic nature of in vivo protein corona is suggested to be considered in future works.

Furthermore, the separation of SC proteins remains a significant challenge. Due to its low binding affinity and high dissociation rate, the SC is prone to loss and contamination during separation, which can compromise compositional analysis and functional interpretation. To better elucidate its role in NP fate, future studies should consider developing mild separation strategies and in situ analytical techniques to more accurately capture PC composition and dynamics under physiological conditions.

## 4 Experimental Section

### Materials

Zymosan from *Saccharomyces cerevisiae* (Z4250) and phorbol 12‐myristate 13‐acetate (P8139) were purchased from Sigma‐Aldrich. Human C3a ELISA kits (EH2734) and human C5a ELISA kits (EH0096) were obtained from Wuhan Fine Biotechnology Co., Ltd, China. Sheep erythrocytes (4%, S9450) and rabbit polyclonal anti‐sheep antibody (1:4000, H8360) were purchased from Solar‐Bio Co., Ltd, China. BCA protein assay kits (P0010) were purchased from Beyotime Biotechnology, China. Precast protein plus gels (Bis‐Tris 4–20%, 36267ES10) were obtained from Yeasen Biotechnology (Shanghai) Co., Ltd, China. Anti‐C3/C3b antibody (ab2000999) was obtained from abcam. Goat anti‐rabbit secondary antibody (31466) was purchased from Thermo Fisher Scientific. Other reagents and chemicals were of analytical grade and used as received unless otherwise stated. Milli‐Q water was used in all experiments.

### Collection of Human Serum

Human serum was obtained from consented healthy volunteers. To minimize the effect of donor‐specific protein alterations, sera from ten donors were pooled and stored in 1 mL aliquots at −80 °C. All human studies were conducted following the ethical guidelines (NO.OUC‐HM‐2022‐001).

### Synthesis of Polystyrene Nanoparticles

The fluorescent polystyrene nanoparticles with carboxy were synthesized by miniemulsion polymerization.^[^ ^42^ ^]^ Briefly, 153.9 mg of acrylic acid, 262.3 mg of hexadecane, 100.7 mg of the initiator 2,2′‐azobis(2‐methylbutyronitrile), and 6.0 mg of FITC were dissolved in 5.9 g of styrene as the oil phase, and 72.8 mg of SDS was added to 24 mL of water to form the aqueous phase; Subsequently, the water phase was poured into the oil phase under stirring at 1000 rpm. The mixture was further stirred at 1000 rpm for 1 h for pre‐emulsification. Next, the pre‐emulsified emulsion was ultrasonicated for 3 min (360 W, 2 s ON, 3 s OFF; JY92‐IIN, SCIENTZ, China) in an ice bath and then stirred overnight at 72 °C for polymerization. Finally, the NP dispersions were purified by dialysis against water for 36 h.

### Synthesis of Magnetic Silica Nanoparticles (SMNs)

SMNs were prepared according to the literature.^[^ ^43^ ^]^ First, Fe_3_O_4_ NPs were synthesized. 21.8 g of ferric chloride and 3.7 g of sodium oleate were dissolved in a mixed solution composed of 8 mL of ethanol, 6 mL of water, and 14 mL of cyclohexane. The mixed solution was heated to 70 °C and reacted for 4 h. Afterward, the reaction mixture was transferred to a separation funnel, and the upper organic layer containing the iron‐oleate complex was collected and washed with water for three times. After washing, cyclohexane was evaporated to obtain waxy iron‐oleate complex. Next, 1.8 g of iron‐oleate complex and 319 µL of oleic acid were dissolved in 12.6 mL of *1*‐octadecene, followed by N_2_ degassing at room temperature for 30 min. Following degassing, the mixture was heated to 320 °C at a constant heating rate of 3.3 °C min^−1^ under N_2_ flow and maintained at this temperature for 30 min. The reaction mixture was then cooled to room temperature and acetone was added to precipitate Fe_3_O_4_ NPs. The precipitate was washed with acetone for five times to obtain pure Fe_3_O_4_ NPs, which were subsequently dispersed in cyclohexane for further utilization.

A reverse microemulsion method was used to prepare SMNs. 500 mg of surfactant Igepal CO‐520 was dissolved in 11 mL of cyclohexane, followed by the addition of 4 mg of Fe_3_O_4_ NPs. The mixed solution was then stirred for 10 min. Subsequently, 100 µL of ammonia hydroxide (30 wt.%) was added and further stirred for another 10 min. Finally, 75 µL of tetramethylsilane was added via the equivalently fractionated drop method (adding 25 µL per 12 h). After the reaction, the mixed solution was centrifuged (10,000 g, 20 min) to obtain SMNs. The SMNs underwent three rounds of ethanol washing through centrifugation and were ultimately dispersed in water.

### Synthesis of Liposomes

The liposomes were prepared using the thin film dispersion method.^[^ ^44^ ^]^ DSPC, DSPE, and cholesterol were dissolved in chloroform at a mass ratio of 2:2:1. A homogeneous dried lipid film was formed at 60 °C in a rotary evaporator and subsequently hydrated in PBS. The liposome suspension was then sonicated (180 W, 1s ON, 1 s OFF; JY92‐IIN, SCIENTZ, China) for 10 min to generate small unilamellar liposomes.

### Characterization of Nanoparticles

The size distribution and zeta potential of NPs were assessed with a particle sizer (Malvern Zetasizer Nano, Malvern, UK) at a fixed scattering angle of 173°. The HENE laser was used as light source (633 nm, 4.0 mW output power). The samples were diluted 100‐fold in PBS at 25 °C for DLS measurements. Morphology of NPs was characterized by using transmission electron microscopy (TEM, JEM‐2100EX, JEOL Ltd., Japan)

### Measurement of Complement Activation by Enzyme‐Linked Immunosorbent Assay (ELISA)

Briefly, the reactions were initiated by incubating the NPs with serum at a volumetric ratio of 1:3 in Eppendorf tubes at 37 °C for 1 h. Then the reactions were stopped by adding PBS containing 20 mM EDTA. Subsequently, the mixtures were centrifuged at 20 000 g for 30 min, and NPs‐induced rises of complement fragments C3a and C5a in the supernatant were determined by commercial ELISA kits. Zymo (1 mg mL^−1^ in water) and pure water were used as positive and negative controls, respectively.

To examine the effect of concentration on complement activation, PS‐COOH was incubated with pool human serum at a final concentration of 0.00572, 0.0286, 0.0572, 0.143, 0.286, and 0.572 m^2^ mL^−1^ for 1 h. Additionally, PS‐COOH (0.143 m^2^ mL^−1^) was incubated with serum for 15, 30, 45, and 60 min to investigate the influence of incubation time on complement activation. The level of C3a and C5a was quantified to determine the effect of NP concentration and incubation time.

To assess the complement activation by different NPs in individual sera from nine donors and their pooled serum, PS‐COOH, SMNs, and Lips (0.143 m^2^ mL^−1^) were incubated with serum for 1 h. To distinguish complement pathways by three NP types, the inhibitor of Ca^2+^‐sensitive pathways EGTA/Mg^2+^ (10 mm EGTA and 10 mm MgCl_2_ final concentration), and the inhibitor of both the alternative pathway and calcium‐sensitive pathways EDTA (10 mm final concentration), were added to sera samples at room temperature for 30 min prior to the experiment. Then PS‐COOH, SMNs, and Lips (0.143 m^2^ mL^−1^) were incubated with serum for 1 h. Finally, the NP‐induced rises of C5a in the supernatant were measured.

### Measurement of Complement Hemolytic Activity

The hemolysis assay was performed to investigate the complement activation of PS‐COOH. Firstly, sheep erythrocytes (2 × 10^8^ cells/mL) and rabbit polyclonal anti‐sheep antibody were separately pre‐incubated in a water bath at 37 °C for 10 min. Subsequently, the cells were mixed with the antibody solution and incubated at 37 °C for 30 min. After incubation, the antibody‐sensitized sheep erythrocytes (EA) were washed twice with PBS and resuspended at a final concentration of 1 × 10^8^ cells/mL in PBS.

Different concentrations of PS‐COOH (0.00572, 0.0286, 0.0572, and 0.572 m^2^ mL^−1^) were incubated with serum at a volumetric ratio of 1:3 at 37 °C for 1 h. Zymo (1 mg mL^−1^ in water) and pure water served as positive and negative controls, respectively. Then the mixtures were centrifuged, and the supernatant of each sample was collected for analysis.

The supernatant was diluted at a volume ratio of 1:4 before conducting the titration reactions. Subsequently, the latter solution was further diluted with different dilution ratios (1:0, 3:1, 3:2, 1:1, 2:3, 1:3, 1:4, 1:5.7, and 1:9). Then, the diluted supernatant (200 µL) was mixed with EA (50 µL). Blank tubes (containing PBS (200 µL) and EA (50 µL)) and 100% lysis tubes (containing only EA (50 µL)) were set up as negative and positive controls, respectively. All Eppendorf tubes were then incubated at 37 °C for 30 min. After incubation, water (200 µL) was added to the 100% lysis tubes. Finally, all tubes were subjected to centrifugation (2000 g, 5 min, 4 °C) to terminate the reactions, and the absorbance (Abs) of the supernatant at 540 nm was measured with a spectrophotometer ((Tecan Spark, Tecan, Switzerland)).

The fractional hemolysis of each tube relative to the 100% lysis tube was calculated as follows: Fractional hemolysis (y) = 100% × (Abs_serum_ – Abs_blank_)/(Abs_100% lysis_ – Abs_blank_). The percentage of complement consumption by NPs was expressed as 100% – y. The volume (in µL) of each supernatant resulting in 50% hemolysis (K) was determined by plotting the log‐log relationship between supernatant volume and [y/(1 – y)], which yielded a linear trace. K corresponded to the x‐axis intercept at 50% hemolysis, for which y/(1 – y) = 1. The CH50 value of the original serum was determined using the formula: CH50 (U mL^−1^) = 4 × (1000/K).

### Preparation of Protein Corona (PC)

As aforementioned for complement activation studies, the PS‐COOH (0.143 m^2^ mL^−1^) was incubated with serum at 37 °C for 1 h. After incubation, the mixtures were centrifuged (20 000 g, 1 h, 4 °C), resulting in the precipitation of PS‐FC. Then, the PS‐FC was washed 3 times with 100 *µL* of PBS to remove loosely bound proteins. The supernatants from the washing steps were separately designated as SN1, SN2, and SN3. The final precipitation was PS‐HC. Then the amounts of PS‐COOH in PS‐FC and PS‐HC were quantified by their fluorescence intensity. For protein identification, the NP‐PC complexes were detached using 100 *µL* of Tris hydrochloride solution containing 2 wt% SDS, followed by incubation at 95 °C for 5 min. The dispersion was then centrifuged, and the resulting supernatant contained desorbed corona proteins. The protein concentrations of PC and in the supernatants of the washing steps were quantified by the BCA protein assay kits according to the manufacturer's instructions.

### Analysis of PC by Sodium Dodecyl Sulfate‐Polyacrylamide Gel Electrophoresis (SDS‐PAGE)

The SDS‐PAGE was used to analyze the proteins in FC, HC, SN1, SN2, and SN3. Briefly, the solution of corona proteins or wash supernatants (20 µL) was mixed with SDS‐PAGE sample buffer and boiled at 100 °C for 10 min. Human serum treated with pure water or Zymo served as a control. The serum proteins were also loaded following the same procedure. The proteins were separated on 4–20% Bis‐Tris gels under a voltage of 150 V for 45 min. Subsequently, the gels were stained with a Coomassie brilliant blue ultrafast staining solution overnight, followed by washing with deionized water until background signals disappeared. Quantification of protein bands was performed using the plot profile tool in Fiji/ImageJ.

### Quantification of C3 Fragments

To compare the amounts of C3 fragments adsorbed on the surface of PS‐COOH, dot blot and western blot were used. For western blot analysis, the corona proteins detached from 2 µg of PS‐COOH were mixed with SDS‐PAGE sample buffer and boiled for 10 min at 100 °C. The corona proteins were separated by SDS‐PAGE (8%) and subsequently transferred onto a nitrocellulose membrane. The membrane was blocked with commercial blocking buffer for 2 h at room temperature. Then the blocked membrane was incubated with the anti‐C3/C3b antibody overnight at 4 °C, followed by incubation with goat anti‐rabbit secondary antibody labeled with horseradish peroxidase for 1 h at room temperature. After washing three times with Tris‐buffered saline containing tween‐20, the proteins were visualized using an enhanced chemiluminescence detection system.

For dot blot analysis, corona proteins were pipetted onto a nitrocellulose membrane and air‐dried for 30 min. Then the nitrocellulose membrane was blocked with commercial blocking buffer for 2 h. After blocked, the membrane was incubated with anti‐C3/C3b antibody overnight at 4 °C and then incubated with goat anti‐rabbit secondary antibody for 1 h. Finally, the protein spots were visualized using an enhanced chemiluminescence detection system.

To further investigate the distribution of C3 fragments in FC and HC, PS‐COOH was incubated with the serum pre‐treated by Zymo (1 mg mL^−1^) or EDTA (10 mm). PBS‐treated serum was used as control sample. Then the PS‐FC and PS‐HC were prepared following the steps outlined in the preparation of PC section. The activated C3 fragments in FC and HC were analyzed by western blot.

For individualized analyses, PS‐COOH, SMNs, and Lips (0.143 m^2^ mL^−1^) were incubated with sera from nine independent donors. FC and HC fractions were isolated from each sample, and the associated C3 activation fragments were quantified by western blot following the procedures described above.

For band and dot blot quantification, the background of 8‐bit greyscale images was subtracted, and integrated densities were measured with Fiji/ImageJ.

### Cell Culture

RAW 264.7 cells were cultured in Dulbecco Modified Eagle Medium (DMEM), and THP‐1 cells were kept in Roswell Park Memorial Institute (RPMI)‐1640 medium. Both media were supplemented with 10% fetal bovine serum (FBS), 100 U mL^−1^ penicillin, and 100 µg mL^−1^ streptomycin. All the cells were cultured at 37 °C under humidified atmosphere with 5% CO_2_ in the air.

### Cellular Uptake of PS‐COOH

The human monocyte cell line THP‐1 was differentiated into macrophages for 3 days before the cellular uptake experiments with NPs. To obtain the dTHP‐1, the THP‐1 (7 × 10^5^) was seeded on a 12‐well plate and treated with phorbol 12‐myristate 13‐acetate (PMA, 100 ng mL^−1^) for 48 h. Then the medium was discarded and the cells were cultured for another 24 h in fresh cell culture medium without PMA.

The RAW 264.7 and dTHP‐1 were seeded at a density of 7 × 10^5^ cells per well in 12‐well plates. After overnight incubation, the medium was changed to serum‐free medium. Then, the prepared PS‐FC and PS‐HC were added to culture media without FBS at a final concentration of 50 µg mL^−1^ and incubated with cells for 0.5, 1, 2, and 4 h at 37 °C. After washing twice with PBS, the cells were collected. Cell fluorescence was measured by flow cytometry (Beckman Moflo XDP, Beckman Coulter, German).

To evaluate the influence of personalized C3 adsorption on macrophage uptake, FC‐ and HC‐coated NPs were prepared using sera from nine individual donors. The FC‐ and HC‐coated NPs (200 µg mL^−1^) derived from each donor serum were incubated with dTHP‐1 cells for 4 h, and uptake was quantified by flow cytometry.

For CLSM, dTHP‐1 cells were cultured in 20 mm confocal culture dishes at 4 × 10^5^ cells/well and treated with 50 µg mL^−1^ PS‐FC or PS‐HC for 4 h. PS‐COOH was labeled with FITC. After exposure, cells were washed three times with PBS to remove unbound PS‐COOH. Then, cells were stained with Lyso‐Tracker Red for lysosomes and Hoechst 33342 for nuclei, according to the manufacturer's instructions. After staining, cells were washed three times with PBS to remove excess dye. Then, 1 mL PBS was added to each dish to keep the cells hydrated. Cells were observed using the CLSM (Leica, TCS SP8, Germany). Hoechst 33342 was excited with a 405 nm laser (blue), and Lyso‐Tracker Red was excited with a 561 nm laser (red).

### Statistical Analysis

All data values were described as the mean values ± standard deviation (S.D.). Triplicated experiments were performed for all experiments in this study. All the statistical analyses were performed using GraphPad Prism 8.0 (GraphPad Software). Statistical differences between the two groups were assessed by Student's *t*‐test. For the comparison of multiple groups, one‐way ANOVA was used. *p < 0.05, **p < 0.01, ***p < 0.001, and ****p < 0.0001.

## Conflict of Interest

The authors declare no conflict of interest.

## Supporting information



Supporting Information

## Data Availability

The data that support the findings of this study are available from the corresponding author upon reasonable request.
