# Supplementary material for: The Distribution of Complement Proteins in Soft and Hard Coronas Impacts Macrophage Uptake of Nanoparticles
Source: Adv Healthc Mater. 2025 Oct 26;15(6):e03534. doi: 10.1002/adhm.202503534 (PMC12892011; doi:10.1002/adhm.202503534)
Supplement: Supplementary file 1 — Supporting Information [file ADHM-15-0-s001.docx]

Supporting Information

The Distribution of Complement Proteins in Soft and Hard Coronas Impacts Macrophage Uptake of Nanoparticles

Ying Qiu, Tianchang He, Chunjie Miao, Xinyang Shi, Yuanyuan Niu, Volker Mailänder, Daniel Crespy, Katharina Landfester,* Shuai Jiang*

Content1

Table S1. Characterization of nanoparticles2

Figure S1. Transmission electron microscope (TEM) images of nanoparticles2

Figure S2. Complement consumption of nanoparticles by homolytic assay3

Figure S3. Characterization of nanoparticles with PC4

Figure S4. Validation of desorption effect of SDS5

Figure S5. Confocal laser scanning microscopy (CLSM) images of nanoparticle internalization6

Figure S6. Complement activation induced by nanoparticles in pooled human7

**Table S1.** Characterization of PS-COOH, SMNs, and Lips in water.


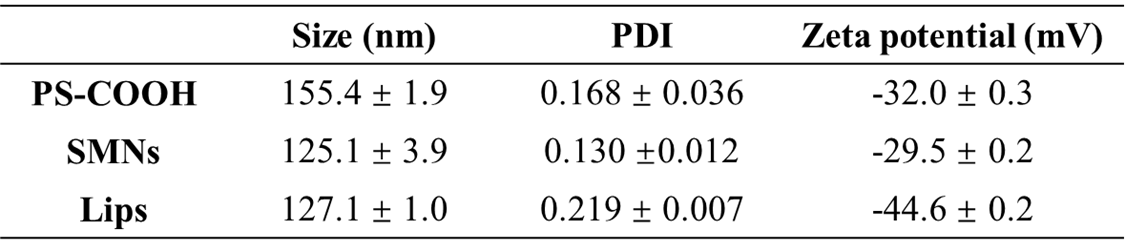


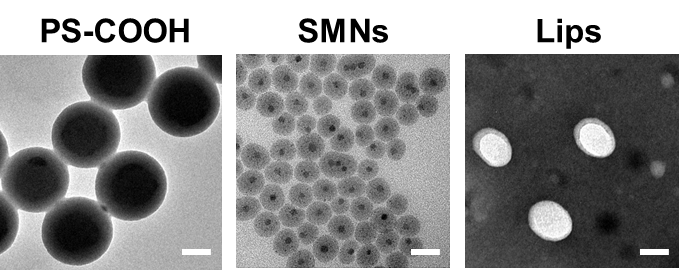


**Figure S1.** Transmission electron microscopy (TEM) images of PS-COOH, SMNs, and Lips. Scale bar: 50 nm.


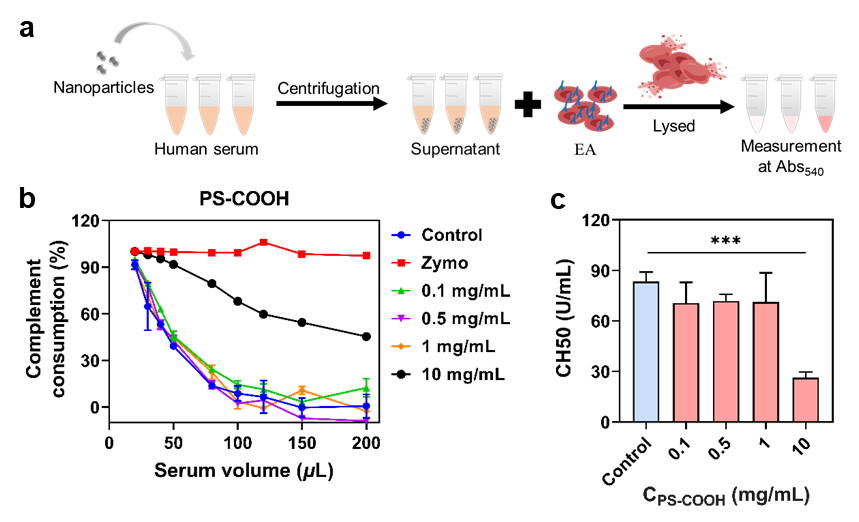


**Figure S2.** Complement consumption of nanoparticles by homolytic assay. (a) Scheme illustrating the procedure of the hemolytic assay. Briefly, nanoparticles were incubated with pooled human serum at 37 ^o^C for 1 h, and the residual complement activity of the treated serum was determined using the EA-based hemolytic assay. Zymo and water were used as positive and negative controls, respectively. (b) Consumption of complement proteins from the serum by PS-COOH. Consumption of complement proteins from the serum by nanoparticles was calculated by 100% - %EA lysis at different dilutions of serum. (c) CH50 of serum after incubation with and PS-COOH. Data are presented as the mean ± standard deviation (n = 3). ****p* < 0.001.


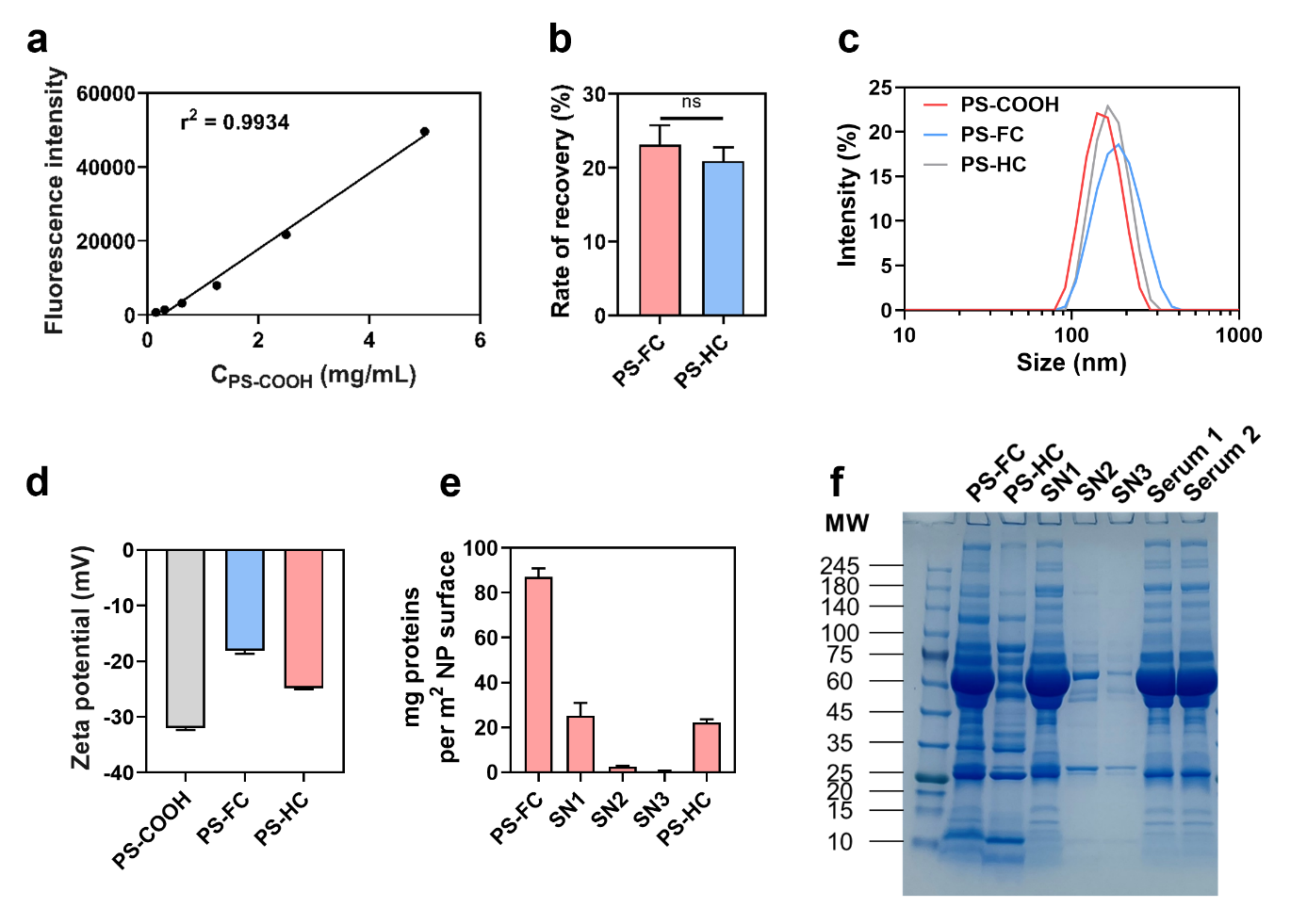


**Figure S3.** Characterization of nanoparticles with PC. (a) Standard curve describing the relationship between concentration and fluorescence intensity of PS-COOH. The standard curve was used to determine the concentration of PS-COOH after incubation with serum. (b) Recovery rate of PS-COOH after incubation with serum. (c) Size distribution and (d) zeta potential of PS-FC and PS-HC. (e) Quantification of protein concentrations in FC, HC, and wash supernatants using BCA assay. (f) SDS-PAGE analysis of FC, HC, and washing supernatants. Data are presented as the mean ± standard deviation (n = 3).


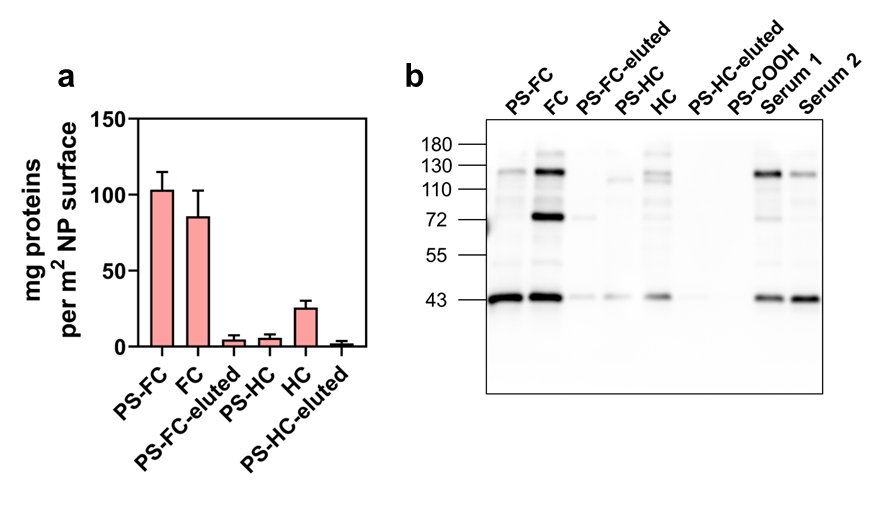


**Figure S4.** Validation of desorption effect of SDS. (a) Concentration of corona proteins of PS-COOH-PC complex before and after desorption determined by BCA assay. (b) Western blot analysis of activated C3 fragments adsorbed on the PS-COOH-PC complexes before and after desorption. Serum 1 represented serum after PBS treatment. Serum 2 represented serum after Zymo treatment. Data are presented as the mean ± standard deviation (n = 3).


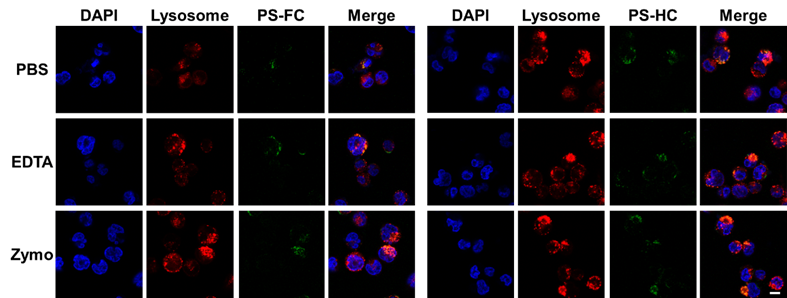


**Figure S5.** Confocal laser scanning microscopy (CLSM) images of PS-FC and PS-HC internalization by dTHP-1 cells at 4 h. Green and red colors correspond to PS-COOH and Lysotracker-labelled acidic endo-lysosomes, respectively. Scale bar: 10 *μ*m


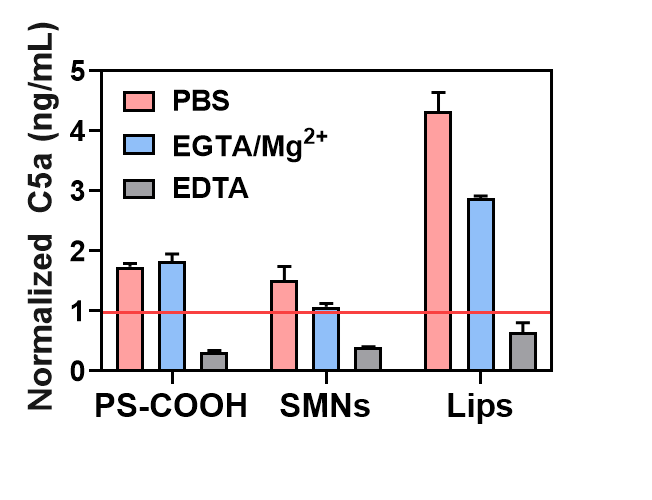


**Figure S6.** Complement activation induced by PS-COOH, SMNs, and Lips in pooled human serum. The pooled serum was prepared by mixing equal volumes of the nine individual serum.
